# The association between uric acid levels and renal function of CKD patients with hyperlipidemia: a sub-analysis of the ASUCA trial

**DOI:** 10.1007/s10157-019-01840-4

**Published:** 2019-12-26

**Authors:** Yoshihiro Kuwabara, Shinji Yasuno, Masato Kasahara, Kenji Ueshima, Kazuwa Nakao

**Affiliations:** 1 Center for Accessing Early Promising Treatment, Kyoto University Hospital, Kyoto, 6068507 Japan; 2 Clinical Research Support Center, Jikei University School of Medicine, Tokyo, Japan; 3 Clinical Research Center, Nara Medical University, Kashihara, Japan; 4 Medical Innovation Center, Kyoto University Graduate School of Medicine, Kyoto, Japan

**Keywords:** Uric acid, Chronic kidney disease, Renal function, Hyperuricemia

## Abstract

**Background:**

The influence of uric acid (UA) on renal function and the significance of UA-lowering therapy are unclear. The purpose of the sub-analysis of the Assessment of Clinical Usefulness in chronic kidney disease patients with Atorvastatin (ASUCA) trial was to evaluate the influence of serum UA levels on renal function in Japanese chronic kidney disease patients with hyperlipidemia.

**Methods:**

Of 344 participants in the ASUCA trial, 279 participants whose UA levels at both baseline and 24 months were available were included. Based on UA level at baseline or mean UA level during the trial period, they were divided into four groups: < 5.0, 5.0–6.0, 6.0–7.0, or ≥ 7.0 mg/dL, irrespective of allocation. Changes in the estimated glomerular filtration rate (eGFR) after 24 months were compared among the groups in relation to baseline or mean UA levels.

**Results:**

For baseline UA levels (< 5.0, 5.0–6.0, 6.0–7.0, or ≥ 7.0 mg/dL), the change in eGFR after 24 months was − 1.32 ± 10.3, − 1.74 ± 8.94, − 2.53 ± 7.34, and − 3.51 ± 9.10 mL/min/1.73 m^2^, respectively. A negative correlation between changes in eGFR after 24 months and baseline UA level was observed with adjustment for confounding factors. The relationship between changes in eGFR and mean UA levels during trial period showed a similar trend.

**Conclusion:**

In CKD patients with dyslipidemia, hyperuricemia was an independent risk factor for CKD progression. An ongoing clinical trial (TARGET-UA, UMIN-ID 000,026,741) may reveal the significance of strict UA-lowering therapy in CKD patients.

**Electronic supplementary material:**

The online version of this article (10.1007/s10157-019-01840-4) contains supplementary material, which is available to authorized users.

## Introduction

Our understanding and interpretation of the significance of the relationship between uric acid (UA) and renal function have changed with time. Although it was widely known in the mid-nineteenth century that gout was associated with chronic kidney disease (CKD) [1], the condition was recognized as being only a marker of renal dysfunction and its involvement in the deterioration renal function was largely unknown [2].

For almost 2 decades, the use of animal models of hyperuricemia without urate crystals has been reported [3–5]. Studies using such models have indicated that excess of UA can itself cause renal dysfunction. Subsequent studies have shown that high levels of UA can also cause endothelial dysfunction, renal tubular damage, glomerulosclerosis, and kidney damage with albuminuria [6–8].

In humans, the association between hyperuricemia and renal dysfunction has been studied primarily through prospective observational research. Iseki and colleagues conducted research on 48,177 healthy Japanese living in Okinawa Prefecture, which revealed that the presence of hyperuricemia (≥ 7.0 mg/dL in males, ≥ 6.0 mg/dL in females) is associated with increased risk of the requirement of initiation of dialysis within 7 years [9]. Bellomo and colleagues examined the renal function of 900 healthy normotensive volunteers over 5 years, and reported a negative correlation between baseline serum UA levels and estimated glomerular filtration rate (eGFR) [10]. Based on several reports including the above-mentioned, it can be concluded that hyperuricemia is involved in the onset of renal dysfunction [11, 12].

Some interventional studies have reported that lowering UA levels through drug therapy has a beneficial effect on renal function. In a study of 54 patients with hyperuricemia combined with CKD, deterioration of renal function was alleviated following administration of allopurinol for 12 months [13]. Allopurinol has also been reported to inhibit renal dysfunction in a study of 113 patients with CKD stage 3 or higher [14]. When allopurinol was discontinued in these patients, deterioration of renal function was observed [15]. These results suggest that therapeutic interventions targeting UA levels may be useful for the protection of renal function.

On the other hand, however, some studies in CKD patients reported that hyperuricemia was not an independent risk factor for CKD progression [16, 17]. The relationship between CKD progression and uric acid levels in CKD patients is still debated.

To further investigate the relationship between UA level and renal function, we analyzed data from the Assessment of Clinical Usefulness in CKD Patients with Atorvastatin (ASUCA) trial, which examined the reno-protective effects of lipid-lowering therapy with atorvastatin in Japanese CKD patients with hyperlipidemia. Using the data of this trial, we determined the relationship between renal function evaluated by eGFR and UA levels at baseline and during the trial period.

## Materials and methods

### Design and participants of the ASUCA trial

This is a post hoc sub-analysis of data from the ASUCA trial (UMIN000001778)—a randomized, open-label, parallel-group comparison trial that examined the effectiveness of lipid-lowering therapy using statin for CKD patients with hyperlipidemia. The protocol and main results have already been reported [18, 19].

The ASUCA trial enrolled 344 Japanese CKD patients with dyslipidemia. The main eligibility criteria at enrollment were as follows: (1) 40–75 years old, (2) not being treated with statin, and (3) urinary protein positive or an eGFR of < 60 mL/min/1.73 m^2^. After screening, 334 patients were randomized into the control group or the atorvastatin group. The treatment period was 24 months and the target low-density lipoprotein cholesterol (LDL-C) level was 100 mg/dL. In the control group, treatment began with diet therapy followed by the administration of drugs other than statin if LDL-C was not sufficiently reduced. The atorvastatin group was treated by administration of atorvastatin in addition to diet therapy. Laboratory tests during a study period were scheduled to be done just before the start of treatment protocol, and 1, 3, 6, 9, 12, 18, and 24 months after the start of treatment protocol.

## Design of the sub-analysis and study population

In this sub-analysis, of 344 participants of the ASUCA trial, 279 participants whose data were available at baseline and after 24 months of treatment were examined, irrespective of allocation. For analysis of relationship between baseline UA levels and eGFR changes, we divided the participants into four groups according to baseline serum UA level: < 5.0 mg/dL, 5.0–6.0 mg/dL, 6.0–7.0 mg/dL, or ≥ 7.0 mg/dL. Next, we analyzed the relationship of renal function with mean UA level by dividing the participants into four groups according to their mean serum UA level during the trial period: < 5.0 mg/dL, 5.0–6.0 mg/dL, 6.0–7.0 mg/dL, and ≥ 7.0 mg/dL.

### Statistical analysis

Continuous variables are expressed as mean values ± standard deviation. Categorical variables are described as percent. Values of two-tailed *p* < 0.05 were considered significant. The primary endpoint was the change in eGFR at 24 months from baseline. One-way analysis of variance (ANOVA) was applied to compare average values among the groups, and linear multiple regression analysis was also performed. In conducting multiple regression analysis, for comparison between four groups based on baseline UA level, sex, age, baseline eGFR, log-transformed urinary albumin excretion (UACR), presence of hypertension, diabetes, use of RAAS inhibitor, use of diuretics, smoking habit, systolic blood pressure level, and allocation were adjusted, and for groups based on mean UA level during the trial period, sex, age, baseline eGFR, log-transformed UACR, baseline UA, presence of hypertension, diabetes, use of RAAS inhibitor, use of diuretics, smoking habit, systolic blood pressure level, and allocation were adjusted. All statistical analyses were performed with EZR (Saitama Medical Center, Jichi Medical University, Saitama, Japan), which is a modified version of R commander designed to incorporate statistical functions frequently used for biostatistics [20].

## Results

The participants’ characteristics according to baseline UA groups are summarized in Table 1. Patients with higher UA tended to be male, and have lower baseline eGFR and more prevalence of RAAS inhibitor use. The eGFR changes over 24 months were − 1.32 ± 10.3, − 1.74 ± 8.94, − 2.53 ± 7.34, and  − 3.51 ± 9.10 mL/min/1.73 m^2^ for the < 5.0 mg/dL, 5.0–6.0 mg/dL, 6.0–7.0 mg/dL, and ≥ 7.0 mg/dL baseline serum UA groups, respectively. When comparing between groups without adjustment, the decrease in eGFR tended to be larger when the baseline serum UA level was higher, although this was not statistically significant. Linear multiple regression analysis (Table 2) adjusted for sex, age, baseline eGFR, log-transformed UACR, presence of hypertension, diabetes, use of RAAS inhibitor, use of diuretics, smoking habit, systolic blood pressure level, and allocation, which indicated that the group with baseline UA levels ≥ 7.0 mg/dL experienced a significantly greater reduction in eGFR compared with the group with baseline UA levels < 5.0 mg/dL.

**Table 1:** Participants’ characteristics between groups based on baseline uric acid level

	~ 5.0 mg/dL	5.0–6.0 mg/dL	6.0–7.0 mg/dL	~ 7.0 mg/dL	*p* value
*n*	42	65	88	84	
Male, *n* (%)	14 (33.3)	34 (52.3)	62 (70.5)	66 (78.6)	< 0.001
Age (years)	63.6 (7.2)	63.5 (8.7)	63.6 (8.0)	62.6 (8.6)	0.83
Allocation (Atorvastatin group), *n* (%)	24 (57.1)	24 (36.9)	46 (52.3)	39 (46.4)	0.150
Disease complication (with duplication)					
Hypertension, *n* (%)	29 (69.0)	51 (78.5)	64 (72.7)	72 (85.7)	0.105
Diabetes, *n* (%)	24 (57.1)	42 (64.6)	57 (64.8)	67 (79.8)	0.85
Baseline eGFR (mL/mm/1.73 m^2^)	58.0 (10.1)	57.3 (11.5)	56.0 (11.5)	51.7 (11.2)	0.004
Baseline uric acid (mg/dL)	4.1 (0.8)	5.6 (0.3)	6.4 (0.3)	8.1 (1.1)	< 0.001
U-Alb (mg/g) creatinine	221.3 (470.4)	250.4 (741.6)	183.2 (413.1)	446.4 (938.3)	0.076
Log-transformed U-Alb (mg/g) creatinine	3.43 (2.06)	3.43 (1.85)	3.52 (1.93)	4.18 (2.09)	0.058
SBP (mmHg)	134.3 (17.5)	133.8 (17.1)	131.9 (14.2)	134.0 (17.1)	0.78
DBP (mmHg)	76.2 (11.7)	77.4 (10.2)	77.1 (9.3)	77.3 (11.6)	0.95
Heart rate (bpm)	70.5 (8.1)	69.5 (11.0)	70.6 (11.2)	73.5 (12.6)	0.139
BMI (kg/m^2^)	25.4 (4.0)	25.5 (3.9)	25.5 (3.2)	26.2 (3.7)	0.52
RAAS inhibitor use, *n* (%)	24 (57.1)	42 (64.6)	57 (64.8)	67 (79.8)	0.039
Diuretics use, *n* (%)	4 (9.5)	7 (10.8)	15 (17.0)	18 (21.4)	0.20
Smoking habits					0.29
Current, *n* (%)	4 (9.5)	6 (9.2)	12 (13.6)	15 (17.9)	
Past, *n* (%)	4 (9.5)	10 (15.4)	17 (19.3)	18 (21.4)	
eGFR change after 24 months (mL/mm/1.73 m^2^)	− 1.3 (10.3)	− 1.7 (8.9)	− 2.5 (7.3)	− 3.5 (9.1)	0.50
SBP at 24 months (mmHg)	128.9 (18.9)	132.1 (14.3)	129.2 (13.2)	133 (15.5)	0.27
RAAS inhibitor use at 24 months, *n* (%)	23 (54.8)	42 (64.6)	56 (63.6)	67 (79.8)	0.021

*U-Alb* urinary albumin excretion, *SBP* systolic blood pressure, *DBP* diastolic blood pressure, *BMI* body mass index, *RAAS* renin–angiotensin aldosterone system, *eGFR* estimated glomerular filtration rate

**Table 2:** Association between serum uric acid level at baseline and change in eGFR after 24 months

	Model 1			Model 2			Model 3		
	*β*	95%CI	*p *value	*β*	95%CI	*p *value	*β*	95%CI	*p *value
~ 5.0 mg/dL	Ref			Ref			Ref		
5.0–6.0 mg/dL	− 1.48	(− 4.57, 1.62)	0.35	− 1.51	(− 4.63, 1.6)	0.34	− 1.76	(− 4.85, 1.34)	0.26
6.0–7.0 mg/dL	− 2.96	(− 5.95, 0.04)	0.053	− 2.96	(− 5.98, 0.05)	0.054	− 2.98	(− 5.97, 0)	0.05
~7.0 mg/dL	− 3.8	(− 6.95, -0.66)	0.018	− 3.79	(− 6.96, − 0.63)	0.019	− 4.14	(− 7.3, − 0.99)	0.01

Model 1: Adjusted for age, sex, allocation, baseline eGFR, log-transformed UACR, RAAS inhibitor use, and the presence of hypertension and of diabetes mellitus

Model 2: Adjusted for age, sex, allocation, baseline eGFR, log-transformed UACR, RAAS inhibitor use, the presence of hypertension and of diabetes mellitus, diuretics use, and smoking habits

Model 3: Adjusted for age, sex, allocation, baseline eGFR, log-transformed UACR, RAAS inhibitor use at 24 months, the presence of diabetes mellitus, diuretics use, smoking habits, and SBP at 24 months

The characteristics of the four groups according to mean UA levels during the trial period were similar to that of baseline UA levels (Table 3). The eGFR changes throughout the trial period were − 0.67 ± 9.94, − 1.86 ± 8.48, − 1.81 ± 7.87, and − 4.45 ± 9.13 mL/min/1.73 m^2^ for groups with mean UA levels of < 5.0 mg/dL, 5.0–6.0 mg/dL, 6.0–7.0 mg/dL, and ≥ 7.0 mg/dL respectively. Higher mean UA levels were found to be associated with a more pronounced decrease in eGFR, although this was not statistically significant (*p* for trend = 0.07). Linear multiple regression analysis (Table 4); adjusted for sex, age, baseline eGFR, log-transformed UACR, baseline UA, presence of hypertension, diabetes, use of RAAS inhibitor, use of diuretics, smoking habit, systolic blood pressure level, and allocation; indicated that decrease in eGFR was significantly larger for the group with mean UA levels of ≥ 7.0 mg/dL than for the group with mean UA < 5.0 mg/dL.

**Table 3:** Patients’ characteristics between groups based on uric acid level during the trial period

	~ 5.0 mg/dL	5.0–6.0 mg/dL	6.0–7.0 mg/dL	~ 7.0 mg/dL	*p* value
*N*	35	69	93	82	
Male, *n* (%)	10 (28.6)	36 (52.2)	63 (67.7)	67 (81.7)	< 0.001
Age (years)	64 (7.7)	64.4 (7.2)	63.4 (8.6)	61.9 (8.7)	0.27
Allocation (atorvastatin group), *n* (%)	21 (60.0)	29 (42.0)	39 (41.9)	44 (53.7)	0.144
Disease complication (with duplication)					
Hypertension, *n *(%)	21 (60.0)	55 (79.7)	71 (76.3)	69 (84.1)	0.037
Diabetes, *n* (%)	14 (40.0)	26 (37.7)	32 (34.4)	40 (48.8)	0.26
Baseline eGFR (mL/mm/1.73 m^2^)	62.3 (12.3)	55.9 (9.8)	54.5 (10.3)	52.7 (12.4)	< 0.001
Baseline uric acid (mg/dL)	4.3 (0.9)	5.7 (0.8)	6.4 (0.9)	7.8 (1.4)	< 0.001
U-Alb (mg/g) creatinine	99.5 (229)	189.4 (439)	222.6 (615.9)	509.9 (989.3)	0.004
Log-transformed U-Alb (mg/g) creatinine	3.01 (1.7)	3.6 (1.8)	3.44 (1.92)	4.31 (2.21)	0.004
SBP (mmHg)	128.4 (14.7)	135.5 (17.7)	133.1 (16.1)	133.9 (15.6)	0.20
DPB (mmHg)	71.5 (10.0)	79.1 (10.0)	78.1 (10.2)	76.7 (11.0)	0.004
Heart rate (bpm)	68.3 (8.3)	70.2 (9.8)	72.2 (11.5)	72.3 (13.0)	0.24
BMI (kg/m^2^)	23.9 (3.6)	26 (3.5)	25.6 (3.7)	26.3 (3.5)	0.012
RAAS inhibitor use, *n* (%)	18 (51.4)	49 (71.0)	60 (64.5)	63 (76.8)	0.043
Diuretics use, *n* (%)	1 (2.9)	9 (13.0)	13 (14.0)	21 (25.6)	0.012
Smoking habits					0.056
Current, *n* (%)	1 (2.9)	8 (11.6)	11 (11.8)	17 (20.7)	
Past, *n* (%)	3 (8.6)	11 (15.9)	18 (19.8)	17 (20.7)	
eGFR change after 24 months (mL/mm/1.73 m^2^)	− 0.7 (9.9)	− 1.9 (8.5)	− 1.8 (7.9)	− 4.5 (9.1)	0.086
eGFR % change after 24 months (%)	− 0.16 (15.3)	− 2.90 (16.3)	− 2.79 (15.7)	− 8.99 (19.9)	0.026
SBP at 24 months (mmHg)	129.8 (17.8)	130.9 (15.8)	130.8 (13.6)	131.7 (15.2)	0.94
RAAS inhibitor use at 24 months, *n* (%)	17 (48.6)	47 (68.1)	62 (66.7)	62 (75.6)	0.042

*U-Alb* urinary albumin excretion, *SBP* systolic blood pressure, *DBP* diastolic blood pressure, *BMI* body mass index, *RAAS* renin–angiotensin aldosterone system, *eGFR* estimated glomerular filtration rate

**Table 4:** Association between serum uric acid level during the trial period at baseline and change in eGFR after 24 months

	Model 1			Model 2			Model 3		
	*β*	95%CI	*p *value	*β*	95%CI	*p *value	*β*	95%CI	*p *value
~ 5.0 mg/dL	Ref			Ref			Ref		
5.0–6.0 mg/dL	− 2.81	(− 6.3, 0.69)	0.115	− 2.79	(− 6.32, 0.73)	0.120	− 2.84	(− 6.32, 0.65)	0.110
6.0–7.0 mg/dL	− 4.01	(− 7.72, − 0.31)	0.034	− 3.99	(− 7.73, -0.25)	0.037	− 4	(− 7.69, − 0.31)	0.034
~ 7.0 mg/dL	− 5.84	(− 10.41, − 1.28)	0.012	− 5.79	(− 10.41, − 1.16)	0.014	− 5.78	(− 10.34, − 1.22)	0.013

Model 1: Adjusted for age, sex, allocation, baseline UA, baseline eGFR, log-transformed U-Alb, RAAS inhibitor use, and the presence of hypertension and of diabetes mellitus

Model 2: Adjusted for age, sex, allocation, baseline UA, baseline eGFR, log-transformed UACR, RAAS inhibitor use, the presence of hypertension and of diabetes mellitus, diuretics use, and smoking habits

Model 3: Adjusted for age, sex, allocation, baseline UA, baseline eGFR, log-transformed UACR, RAAS inhibitor use at 24 months, the presence of diabetes mellitus, diuretics use, smoking habits, and SBP at 24 months

Furthermore, because the distribution of serum uric acid levels is different between men and women, multivariable regression analyses were also stratified by sex. Since the number of cases in each patient group is relatively small, stratified analysis by sex is provided as supplementary data for exploratory studies (Tables S1 and S2 in the Supplementary Appendix).

## Discussion

This study revealed a negative correlation between baseline serum UA levels and renal function after 24 months of lipid-lowering treatment in Japanese CKD patients with hyperlipidemia. When baseline UA levels were below 5.0 mg/dL, reduced deterioration of renal function was observed throughout the trial period compared with patients whose baseline levels were ≥ 7.0 mg/dL, even after adjustment for age, sex, baseline eGFR, and systolic blood pressure. Mean serum UA levels during the trial period were also found to be associated with renal function. When mean UA levels were < 5.0 mg/dL, renal function was preserved compared with that of patients whose mean UA levels were ≥ 7.0 mg/dL, even after adjustment for possible confounders.

Several studies have used animal models to estimate the mechanisms in which low UA levels protect renal function. Sanchez-Lozada et al. reported that increased UA levels lead to increased oxidative stress, impairment of vascular endothelial function, and increased renal vascular resistance resulting in systemic or glomerular hypertension [21–23]. Nakagawa et al. conducted a study using a rat model of hyperuricemia, and demonstrated that this condition was associated with glomerular hypertrophy and increased urinary albumin [6]. From examination of rat kidneys, Ryu et al. [7] reported that high levels of UA can contribute to the progression of interstitial fibrosis via the epithelial-to-mesenchymal transition (EMT) of renal tubular cells. According to Kang et al. [24], hyperuricemia can promote glomerular sclerosis, and it has also been reported this condition inhibits the function of endothelial cells [8]. These mechanisms may provide some explanation for the results of our study.

UA is also known to have antioxidant activity and to be involved in the type 2 immune response, and recent attention has been paid to its potential protective activity toward living bodies and organs [25, 26]. Therefore, extreme hypouricemia and excessive UA reduction therapy may be associated with poor prognosis. Hayashino et al. [27] examined the effects of baseline UA levels on the onset or progression of renal dysfunction in a cohort of Japanese diabetic patients. Patients were divided according to the quartile in which their baseline UA level, and the hazard ratio of development of overt albuminuria was higher in the first, third, and fourth quartiles compared with the second quartile. This suggests the possibility of a J curve-like correlation between UA levels and the progression of CKD. However, in the present study, the decrease in renal function was less pronounced in the group with low UA levels than that observed for groups with higher UA levels during the trial period. This suggests that when serum UA levels are < 5.0 mg/dL—which is lower than the target level in the current general clinical practice—renal function may be protected.

In a report by Hayashino et al., low UA levels (within the first quartile; average = 3.6 mg/dL, quartile range = 3.2–4.0) were associated with a higher risk of developing overt proteinuria. Our data showed that, in the group with lowest UA, an average baseline UA level was 4.1 mg/dL (interquartile range = 4.0–4.9), and an average UA value during the trial period was 4.2 (interquartile range = 3.9–4.7). Therefore, the effect of UA levels < 4.0 mg/dL on renal function has not been sufficiently examined. Extremely low UA levels may be related to poor renal function, but further studies are needed to confirm this.

A randomized-controlled trial of topiroxostat in Japanese CKD patients with hyperuricemia is currently ongoing (TARGET-UA, UMIN-ID 000026741) to investigate whether the administration of UA-lowering therapy, adapted to achieve a strict therapeutic management target level, could represent an approach with which to improve renal-function protection. It is expected that the optimal method of UA management will be clarified for patients with CKD based on the results of these studies.

Our research has several limitations. First, it is a post hoc analysis, and therefore, the possibility of uncontrolled confounding factors remains. For example, the status of using uric acid-lowering drugs was unknown. Second, an intervention study is necessary to demonstrate whether the target value presented at this time is useful for therapeutic intervention in CKD patients. Third, the participants of this study were CKD patients with hyperlipidemia, and the underlying diseases of CKD were mainly diabetes and hypertension. It is, therefore, unclear whether our results will apply to general CKD patients. Finally, because the sample size in this study was relatively small, the relationship between changes in renal function and serum uric acid levels may not be fully accurately assessed.

## Conclusions

In CKD patients with dyslipidemia, increased baseline serum UA levels and mean levels throughout the trial period were associated with prognosis of renal function, respectively. Further studies are needed to determine whether UA level lower than that currently targeted in general clinical practice is beneficial for the renal function.

## Electronic supplementary material

Below is the link to the electronic supplementary material.

Supplementary file1 (DOCX 22 kb)
