# Supplementary material for: The association between uric acid levels and renal function of CKD patients with hyperlipidemia: a sub-analysis of the ASUCA trial
Source: Clin Exp Nephrol. 2019 Dec 26;24(5):420–6. doi: 10.1007/s10157-019-01840-4 (PMC7174259; doi:10.1007/s10157-019-01840-4)
Supplement: Supplementary file 1 — Supplementary file1 (DOCX 22 kb) [file 10157_2019_1840_MOESM1_ESM.docx]

Table S1. Association between serum uric acid level at baseline and change in eGFR after 24 months stratified by sex.

| Male |  | | |  | | |  | | |
| --- | --- | --- | --- | --- | --- | --- | --- | --- | --- |
|  | Model 1 | | | Model 2 | | | Model 3 | | |
|  | β | 98%CI | p-value | β | 98%CI | p-value | β | 98%CI | p-value |
| ~5.0 mg/dL | Ref. |  |  | Ref. |  |  | Ref. |  |  |
| 5.0~6.0 mg/dL | -3.72 | (-8.84, 1.39) | 0.153 | -3.73 | (-8.9, 1.43) | 0.155 | -3.98 | (-9.1, 1.14) | 0.126 |
| 6.0~7.0 mg/dL | -4.42 | (-9.23, 0.4) | 0.072 | -4.5 | (-9.36, 0.36) | 0.069 | -4.69 | (-9.46, 0.07) | 0.054 |
| 7.0 mg/dL~ | -5.9 | (-10.78, -1.01) | 0.018 | -6.03 | (-10.97, -1.08) | 0.017 | -6.41 | (-11.27, -1.54) | 0.010 |
|  |  |  |  |  |  |  |  |  |  |
| Female |  |  |  |  |  |  |  |  |  |
|  | Model 1 | | | Model 2 | | | Model 3 | | |
|  | β | 98%CI | p-value | β | 98%CI | p-value | β | 98%CI | p-value |
| ~5.0 mg/dL | Ref. |  |  | Ref. |  |  | Ref. |  |  |
| 5.0~6.0 mg/dL | 0.14 | (-3.79, 4.06) | 0.94 | 0.06 | (-3.96, 4.08) | 0.98 | -0.23 | (-4.3, 3.84) | 0.91 |
| 6.0~7.0 mg/dL | -1.98 | (-6.13, 2.18) | 0.34 | -2.07 | (-6.28, 2.13) | 0.33 | -2.4 | (-6.63, 1.82) | 0.26 |
| 7.0 mg/dL~ | -1.6 | (-6.32, 3.12) | 0.50 | -2.37 | (-7.22, 2.49) | 0.34 | -2.98 | (-7.93, 1.97) | 0.23 |

Model 1: Adjusted for age, allocation, baseline eGFR, Log-transformed UACR, RAAS inhibitor use, and the presence of hypertension and of diabetes mellitus.

Model 2: Adjusted for age, allocation, baseline eGFR, Log-transformed UACR, RAAS inhibitor use, the presence of hypertension and of diabetes mellitus, diuretics use, and smoking habits.

Model 3: Adjusted for age, allocation, baseline eGFR, Log-transformed UACR, RAAS inhibitor use at 24 months, the presence of diabetes mellitus, diuretics use, smoking habits, and SBP at 24 months.

Table S2. Association between serum uric acid level during the trial and change in eGFR after 24 months stratified by sex.

| Male |  | | |  | | |  | | |
| --- | --- | --- | --- | --- | --- | --- | --- | --- | --- |
|  | Model 1 | | | Model 2 | | | Model 3 | | |
|  | β | 98%CI | p-value | β | 98%CI | p-value | β | 98%CI | p-value |
| ~5.0 mg/dL | Ref. |  |  | Ref. |  |  | Ref. |  |  |
| 5.0~6.0 mg/dL | -0.95 | (-7.11, 5.21) | 0.76 | -1.03 | (-7.26, 5.19) | 0.74 | -1.24 | (-7.43, 4.95) | 0.69 |
| 6.0~7.0 mg/dL | -3.17 | (-9.36, 3.03) | 0.31 | -3.28 | (-9.55, 2.98) | 0.30 | -3.38 | (-9.61, 2.84) | 0.28 |
| 7.0 mg/dL~ | -3.82 | (-10.79, 3.15) | 0.28 | -4.06 | (-11.14, 3.02) | 0.26 | -3.98 | (-11.02, 3.07) | 0.27 |
|  |  |  |  |  |  |  |  |  |  |
| Female |  |  |  |  |  |  |  |  |  |
|  | Model 1 | | | Model 2 | | | Model 3 | | |
|  | β | 98%CI | p-value | β | 98%CI | p-value | β | 98%CI | p-value |
| ~5.0 mg/dL | Ref. |  |  | Ref. |  |  | Ref. |  |  |
| 5.0~6.0 mg/dL | -3.96 | (-8.46, 0.55) | 0.084 | -3.09 | (-7.81, 1.62) | 0.195 | -3.68 | (-8.27, 0.91) | 0.114 |
| 6.0~7.0 mg/dL | -4.11 | (-9.25, 1.03) | 0.116 | -3.17 | (-8.56, 2.23) | 0.25 | -4 | (-9.22, 1.23) | 0.132 |
| 7.0 mg/dL~ | -9.31 | (-17.1, -1.52) | 0.020 | -8.07 | (-16.17, 0.03) | 0.051 | -8.84 | (-16.76, -0.93) | 0.029 |

Model 1: Adjusted for age, allocation, baseline eGFR, baseline UA, Log-transformed UACR, RAAS inhibitor use, and the presence of hypertension and of diabetes mellitus.

Model 2: Adjusted for age, allocation, baseline eGFR, baseline UA, Log-transformed UACR, RAAS inhibitor use, the presence of hypertension and of diabetes mellitus, diuretics use, and smoking habits.

Model 3: Adjusted for age, allocation, baseline eGFR, baseline UA, Log-transformed UACR, RAAS inhibitor use at 24 months, the presence of diabetes mellitus, diuretics use, smoking habits, and SBP at 24 months.
